# Associations of Stress and Appetite Hormones with Binge Eating in Females with Anorexia Nervosa after Weight Restoration: A Longitudinal Study

**DOI:** 10.3390/jpm11101020

**Published:** 2021-10-12

**Authors:** Ya-Ke Wu, Kimberly A. Brownley, Anna M. Bardone-Cone, Cynthia M. Bulik, Jessica H. Baker

**Affiliations:** 1 School of Nursing, University of North Carolina at Chapel Hill, Chapel Hill, NC 27599, USA; yakew@email.unc.edu; 2 Department of Psychiatry, University of North Carolina at Chapel Hill, Chapel Hill, NC 27599, USA; kim_brownley@med.unc.edu (K.A.B.); cynthia_bulik@med.unc.edu (C.M.B.); 3 Department of Psychology and Neuroscience, University of North Carolina at Chapel Hill, Chapel Hill, NC 27599, USA; bardonecone@unc.edu; 4 Department of Medical Epidemiology and Biostatistics, Karolinska Institutet, 17177 Solna, Sweden; 5 Department of Nutrition, University of North Carolina at Chapel Hill, Chapel Hill, NC 27599, USA

**Keywords:** loss of control eating, renourishment, stress hormones, appetite hormones

## Abstract

Binge eating is a transdiagnostic eating disorder symptom that can occur in patients with anorexia nervosa (AN), persisting after weight restoration, and impeding their recovery. However, little is known about the biological predictors of binge eating after AN weight restoration. The goals of this exploratory study of 73 females with AN were: (1) to examine changes in cortisol, the adrenocorticotropic hormone, norepinephrine, ghrelin (total and active), and leptin levels across the admission, discharge, and 3 months post-discharge from the inpatient AN weight restoration; and (2) to determine whether the target hormones were associated with objective or subjective binge eating (OBE or SBE). The participants completed the self-reported Eating Disorder Examination Questionnaire, Beck Anxiety Inventory, and Beck Depression Inventory-II, and provided fasting whole blood samples for hormone assays. The results showed significant changes in body mass index (BMI), cortisol, total ghrelin, and leptin levels over the three time points. The cortisol levels at admission and discharge were significantly associated with the number of SBE episodes at 3 months post-discharge. Findings suggest the need to replicate and confirm the role of cortisol in predicting the emergence of SBE and uncover the mechanisms underlying SBE and cortisol to prevent SBE and its negative consequences.

## 1. Introduction

Anorexia nervosa (AN) is an eating disorder defined by a restriction of energy intake leading to a significantly low body weight [1]. Patients with AN often display anxiety toward food, fear weight gain, and fail to recognize the seriousness of their low body weight [2]. Weight restoration is a major component of treatment for AN [3] and, for some patients, can involve hospital-based inpatient renourishment [3]. Binge eating is a transdiagnostic eating disorder symptom that occurs in some individuals with AN (i.e., AN binge-eating/purging subtype) [4]. When present, binge eating can persist after weight restoration and affect the recovery from AN [5]. Compared with patients with AN who do not binge eat, those who binge eat frequently experience more adverse consequences (e.g., increased food addiction and compulsive exercise) that may delay recovery and prolong the duration of illness [6,7,8,9]. Despite the potential consequences of binge eating on the treatment outcome and recovery, little is known about the prospective predictors of binge eating in patients with AN after weight restoration.

Generally, binge eating can be classified as: (1) objective binge eating (OBE), characterized by consuming an unusually large amount of food that is definitely larger than what most people would eat under similar circumstances, combined with a sense of loss of control over eating (i.e., being compelled to eat or unable to resist or stop eating) [1]; or (2) subjective binge eating (SBE), also characterized by a sense of loss of control over eating, but the quantity of food consumed is not objectively large by social comparison, although the individual experiences it as being a large or excessive amount [10,11]. Though OBE is a key diagnostic criterion for binge eating as observed in bulimia nervosa, binge-eating disorder, and AN binge-eating/purging subtype, loss of control while eating, regardless of the amount of food consumed, is shown to be a better predictor of adverse health outcomes in patients with eating disorders than consuming unusually large amounts of food [11]. Indeed, SBE frequency predicts poorer recovery rates in patients with bulimia nervosa or binge-eating disorder [12] and, compared with individuals with OBE, those with SBE are more likely to experience persistent eating disorder symptoms and psychopathology such as weight or shape concerns, diuretic use, depressive symptoms, anxiety, and social avoidance [13].

Binge eating is a complex behavior influenced by biological, psychological, and social factors [14]. One psychological factor that contributes to the development of binge eating is psychological stress [15,16,17,18]. Psychological stress, while a broad term, generally involves interactions between individuals and their environments which may exceed the adaptive capacity of the individual, resulting in psychological and biological changes which may place individuals at risk of disease [19,20]. For example, the perceived stress measured by the Perceived Stress Scale is associated with an increased risk of OBE in adults from a community sample [21]. A mixed body of evidence suggests that people who binge eat report that food buffers negative emotions by providing comfort and distraction [22,23]. The weight restoration treatment for patients with AN in a hospital-based inpatient setting is often reported as an intense and stressful experience [24]. To achieve weight gain goals for AN, caloric intake can be up to 4000 kcal per day during nutritional rehabilitation [25]. Even though the portion of each meal consumed by AN patients during inpatient renourishment may not be objectively large, since the meals often contain energy-dense foods, patients with AN may be at risk of developing binge eating when consuming a high caloric diet daily in order to achieve target weight goals [26]. In addition, their experiences on an inpatient unit could foster dependence and isolation, and patients may have difficulty adjusting to life outside of the hospital after discharge [27]. Thus, the various aspects of weight restoration on an inpatient unit, including the psychological stress and the high caloric intake, could increase the risk of binge eating after discharge.

Stress hormones may reveal mechanisms underlying binge eating as they relate to the experience of psychological stress. When such a stressor is encountered (e.g., weight restoration treatment on an inpatient unit), norepinephrine (NE) is released from the adrenal glands to help the body respond to stress (i.e., the fight or flight response) [28]. Stress activates the release of the corticotropin-releasing hormone (CRH) from the hypothalamus which triggers the release of the adrenocorticotropic hormone (ACTH), thereby causing cortisol to be released from the adrenal cortex [16]. The prolonged exposure to elevated cortisol levels may dictate eating behaviors such as an increased appetite and food consumption, leading to an increased risk of binge eating [29].

Weight restoration also changes the appetite hormone ghrelin and leptin levels in patients with AN [30,31,32,33]. Renourishment induced a 14% increase in body mass index (BMI) in patients with AN and led to a 25% decrease in circulating ghrelin levels [30]. Fasting ghrelin levels were also higher in untreated AN patients with a binge-eating/purging subtype than in restrictive subtype [34]. Lower leptin levels were found in AN patients with a low body weight compared with the controls [33]. After weight restoration, leptin levels in patients with AN were higher than they were before weight restoration due to their increased body fat mass [35]. Previous studies show that ghrelin and leptin are associated with binge eating in patients with bulimia nervosa and binge-eating disorder [36,37]; however, it remains unclear if the ghrelin and leptin levels observed during renourishment are associated with binge eating after weight restoration in AN. Collectively, cortisol, ACTH, NE, ghrelin, and leptin are reasonable candidates as specific biological markers associated with the emergence of binge eating in AN patients after weight restoration. Identifying reliable biological predictors of binge eating after weight restoration in AN patients could be important for developing personalized renourishment programs and informing post-weight restoration treatment strategies.

Thus, the aim of this exploratory secondary data analysis study was to examine whether stress and appetite hormones could prospectively predict OBE and SBE among females (age ≥ 15) with AN after weight restoration treatment. We collected data at three time points: admission, discharge (i.e., when the patient reaches approximately 80–85% of the ideal body weight), and 3 months post-discharge. The specific aims were to: (1) explore the levels of cortisol, ACTH, NE, ghrelin, and leptin at the three time points; (2) determine the associations between the stress and appetite hormone levels at admission and discharge with OBE and SBE at 3 months post-discharge; (3) examine whether the change in the stress and appetite hormone levels from admission to discharge were associated with OBE and SBE at 3 months post-discharge. Anxiety and depression levels were measured as potentially confounding factors because these psychological factors may increase the risk of binge eating [21].

## 2. Materials and Methods

### 2.1. Participants

This overarching study included 75 females who were recruited from an academic inpatient eating disorder treatment program and met the following criteria: (1) assessed within five days of admission to the inpatient program; (2) met the Diagnostic and Statistical Manual of Mental Disorders, Fourth or Fifth Edition (DSM-IV or −5) criteria for AN; (3) were at least 15 years old. AN diagnosis and subtype were determined at admission by a psychiatrist and the treatment team via structured clinical interviewa and the results were documented in patients’ medical records. Exclusion criteria were: (1) pregnant; (2) severe infections (e.g., urinary tract infection); (3) received an immunization in the last 10 days. Because the majority of admissions to the inpatient program were female, only females were included in the study. Participants were approached consecutively as admitted for treatment. Two women consented but later withdrew from the study. Therefore, a total of N = 73 women had data available for inclusion in the current study. For inclusion in the final analysis sample, we did not require data which were 100% complete. All participants provided informed consent. The study was conducted in accordance with the Declaration of Helsinki, and the protocol was approved by the Ethics Committee of the University of North Carolina at Chapel Hill (IRB number 13-1842).

### 2.2. Measurement

#### 2.2.1. Demographics Variables

Participants’ age, age of AN onset, and AN diagnosis and subtype (i.e., binge-eating/purging or restrictive subtype) from the semi-structured clinical interview conducted were obtained from medical records.

#### 2.2.2. Anthropometric Measurements

Height and weight were obtained from medical records at admission and discharge as this was completed on the inpatient unit by nursing staff. At 3 months post-discharge, patients were weighed by interviewers if they attended an in-person study visit and those unable to attend an in-person study visit (e.g., did not live locally) provided weight from a healthcare provider or self-reported their current weight (*n* = 18). BMI was computed as weight in kilograms divided by height in meters squared. The percentage of ideal body weight (%IBW) was also calculated as weight in kilograms multiplied by 100 then divided by the ideal body weight in kilograms (IBW, body height (m^2^) × 21.5) [38].

#### 2.2.3. Biochemical Measurements

Whole blood samples for cortisol, ACTH, NE, active and total ghrelin, and leptin levels were collected in the morning after an overnight fast at admission, discharge, and 3 months post-discharge for those participants able to attend in-person appointments. Samples were immediately transported to the UNC Molecular Biology Research laboratory and frozen at −80 °C until assayed at the UNC Endocrine laboratory using commercially available radioimmunoassay kits. Cortisol levels were assessed by high-sensitivity enzyme immunoassay kits (standard range: 1–60 ug/dL, sensitivity: 0.07 ug/dL). ACTH levels were assessed by radioimmunoassay (RIA) kits (standard range: 7–925 pg/mL, sensitivity: 5 pg/mL). NE levels were assessed by enzyme-linked immunosorbent assay (ELISA) kits (standard range: 5–1000 ng/mL. sensitivity: 0.1 ng/mL). Total and active ghrelin were both measured in this study because the active form of ghrelin could cross the gut–brain barrier and communicate with the brain to play a larger part in feeding behavior compared with nonacylated (des-acyl) ghrelin [39]. Active and total ghrelin levels were assessed by the RIA kits (standard range: active ghrelin: 10–2000 pg/mL; total ghrelin: 100–10,000 pg/mL; sensitivity: 10 pg/mL). Leptin levels were assessed by the ELISA kits (0.5–100 ng/mL; sensitivity: 0.5 ng/mL). All hormone samples were analyzed in duplicate. The intra-assay and inter-assay coefficients of variation of these assays were <5 percent.

#### 2.2.4. Binge Eating

The Eating Disorder Examination Questionnaire (EDE-Q) [40,41] was used at the three time points to measure eating disorder symptoms during the past four weeks, including OBE and SBE. OBE was defined as the number of self-reported episodes of consuming objectively large quantities of food with a sense of loss of control when eating in the past four weeks. SBE was defined as the number of self-reported episodes of eating in which one had a sense of loss of control but did not eat an objectively large amount of food in the past four weeks. The EDE-Q, self-reported OBE frequency was significantly correlated with the Eating Disorder Examination (i.e., a diagnostic interview for eating disorders) OBE frequency [41].

#### 2.2.5. Potential Covariates

*Anxiety.* Anxiety was measured at the three time points using the Beck Anxiety Inventory, a 21-item instrument with a 4-point scale ranging from 0 (not at all) to 3 (severe) for anxiety level during the past week [42]. The inventory included assessment of symptoms such as nervousness, dizziness, and inability to relax [43]. The total score ranges from 0 to 63, with higher scores indicative of more anxiety symptoms [42]. Cronbach’s alpha for the total score was 0.93 in the present study.

*Depression.* Depressive symptoms during the past two weeks were assessed at the three time points using the Beck Depression Inventory-II, a 21-item instrument [44]. Each item included four response options from 0 (symptom absent) to 3 (severe symptoms) indicating increasingly severe levels of depression [45]. The total score ranged from 0 to 63, with higher scores indicative of a greater severity to depression [45]. Cronbach’s alpha for the overall scale was 0.95 in the present study.

All self-reported questionnaires were completed via online survey. Therefore, participants had the opportunity to complete the study questionnaires from any location.

### 2.3. Statistical Analysis

Descriptive statistics (mean and standard deviation [SD], or frequency and percentage, as appropriate) for demographic variables, BMI, hormones, OBE and SBE episodes during the past four weeks, and scores on the anxiety and depression questionnaires were computed. Pearson’s correlation analysis was used to determine the initial associations between continuous variables.

For aim 1 (i.e., exploring the change in the stress and appetite hormone levels across the three time points), because our continuous data violated the normality assumption, we used the Friedman test for non-parametric repeated measures ANOVA to compare the means of cortisol, ACTH, NE, active ghrelin, total ghrelin, and leptin levels across three time points. The Wilcoxon signed-rank test with Bonferroni adjustment was used for the post hoc comparisons. Additionally, we used this same approach to examine change across three time points of BMI, OBE and SBE episodes, anxiety, and depression.

For aim 2 (i.e., determining the associations of the stress and appetite hormone levels at admission and discharge with OBE and SBE at 3 months post-discharge) and aim 3 (i.e., examining whether the change in the stress and appetite hormone levels from admission to discharge was associated with OBE and SBE at 3 months post-discharge), multiple linear regression was used to identify the associations of cortisol, ACTH, NE, active and total ghrelin, and leptin (predictors) with OBE and SBE episodes (outcomes). Continuous variables (i.e., the demographic variables, anxiety, and depression levels) that were significantly correlated with OBE or SBE episodes at 3 months post-discharge were included as covariates. We controlled for pre-treatment OBE and SBE (i.e., the number of OBE and SBE episodes during the four weeks prior to admission) in our regression models. All data were analyzed using the most recent version of SPSS 26 [46]. *p*-values of 0.05 or less were considered statistically significant. Because we considered this an exploratory study, we chose a priori not to correct for multiple testing in our regression models.

## 3. Results

### 3.1. Characteristics of Participants

Table 1 presents the participant characteristics and the results of the repeated-measures ANOVA across admission, discharge, and 3 months post-discharge. The participants’ age ranged from 15 to 77 years old. Approximately 15% (*n* = 11) of the participants were younger than 18 years old. The mean (±SD) number of days spent on the inpatient unit was 29.0 ± 17.8 days, ranging from 3 to 78 days. With regard to the AN subtype, 54.79% (*n* = 40) of participants were classified as binge-eating/purging, and 41.09% (*n* = 30) were classified as restricting. For *n* = 3 participants, a subtype was not coded in the medical record and was missing. At 3 months post-discharge, a total of thirty participants self-reported experiencing binge eating: four participants reported only OBE, sixteen participants reported only SBE, and ten participants reported both OBE and SBE. Among these thirty participants, three reported new onsets of OBE (two participants reported SBE at admission) and four reported new onsets of SBE (one of them reported OBE at admission) at 3 months post-discharge.

### 3.2. Repeated-Measures ANOVA Results

BMI increased significantly across the three time points (Table 1). The mean total ghrelin decreased from admission to discharge but increased from discharge to 3 months post-discharge. A significant increase in leptin was noted over the three time points. Cortisol, anxiety, and depression decreased significantly across the three time points.

### 3.3. Correlation Analysis Results

BMI at admission (r = 0.24, *p* < 0.05) and cortisol levels at admission (r = 0.29, *p* < 0.05) and discharge (r = 0.32, *p* < 0.05) were positively correlated with SBE episodes at 3 months post-discharge. As a result, we included the admission BMI as a covariate in our regression models. No significant correlation was found between age, age of AN onset, anxiety, and depression, and the change in hormone levels with OBE or SBE at 3 months post-discharge, and thus these variables were not included as covariates in the regression models.

### 3.4. Association between Hormone Levels and Binge Eating

The cortisol levels at admission (β = 0.37, *t* = 2.06, *p* = 0.04) and discharge (β = 0.36, *t* = 2.20, *p* = 0.03) were significantly positively associated with the number of SBE episodes self-reported at 3 months post-discharge (Table 2). Conversely, no significant associations were observed between hormone levels at either admission or discharge and the number of self-reported OBE episodes at 3 months post-charge.

### 3.5. Association between Changes of Hormone Levels and Binge Eating

No significant associations were found between changes in hormone levels from admission to discharge and the number of self-reported OBE or SBE episodes at 3 months post-discharge (Table 3).

### 3.6. Post Hoc Analyses

In a post hoc follow up analysis, we combined OBE and SBE at 3 months post-discharge in our regression models to focus on any type of loss of control during eating. The results showed no significant associations between cortisol levels (at admission, discharge, and the change from admission to discharge) and the combination of OBE and SBE at 3 months post-discharge (β = −0.23~0.50, *t* = −0.73~1.93, *p* = 0.06~0.46). Additionally, purging behaviors such as self-induced vomiting and laxative abuse could result in dehydration, decreased blood flow throughout the body, and altered hormone levels [47]. Therefore, we conducted an additional analysis to see whether purging behaviors alone (i.e., the frequency of self-vomiting, laxatives, and diuretics use during the past 4 weeks) at time points 1, 2, and 3 correlated with the levels of hormones at time points 1, 2, and 3. No significant correlation was found between the purging behaviors and the levels of hormones (all *p* > 0.05). We also conducted an additional independent t-test analysis comparing the hormone levels between the participants with AN restricting and binge-eating/purging subtypes. No significant differences were found in any hormone levels between the two groups at any of the time points (all *p* > 0.05).

## 4. Discussion

The present study explored the changes in stress and appetite hormones across inpatient admission, discharge, and 3 months post-discharge in females with AN, and how the admission and discharge levels of hormones and changes in these hormones related to OBE and SBE episodes after weight restoration. Contrasting with our hypothesis that the treatment in an inpatient unit may induce psychological stress, cortisol levels did not significantly increase during inpatient treatment, but rather decreased over time. The hypothalamic-pituitary-adrenal (HPA) axis hyperactivity and high cortisol levels were reported among acute stage AN patients [48]. According to one theory, the increase in cortisol in acute AN was the result of starvation deregulating cortisol feedback inhibition; therefore, this led to an increased CRH release and increased ACTH and cortisol production [49]. During inpatient treatment, starvation decreased with the daily renourishment process, which may account for the decrease in cortisol levels over time.

A significant decrease in total ghrelin was also observed from admission to discharge, while BMI increased. This finding was consistent with Otto and colleagues’ findings in two longitudinal studies that ghrelin levels significantly decreased after weight gain in AN patients [31,32]. Ghrelin was involved in stimulating appetite and increasing food intake [50]. Ghrelin secretion increased during fasting and decreased after eating a normal meal [51,52]. In a recent meta-analysis, all forms of ghrelin were found to increase in patients with acute AN during fasting compared with the healthy controls [53]. The starvation with a low body weight in AN patients at admission might trigger the increase in ghrelin to signal the hypothalamus to stimulate food intake. After the refeeding process, patients were no longer in an acute starvation state, reflected in the lower ghrelin levels at discharge. When patients returned home, they were no longer under a supervised refeeding program and may have a restricted intake again, leading to an increase in ghrelin 3 months after discharge. However, our results showed slight, continuing increases in BMI from discharge to 3 months post-discharge, although at a much slower pace. Thus, the intake restriction and/or increased physical activity after discharge could contribute to the slower increase in BMI after discharge. Despite no statistically significant changes in the active ghrelin levels observed in our patients, active ghrelin levels exhibited a similar pattern to the total ghrelin levels over the three time points.

Furthermore, we found that leptin levels increased significantly from admission to discharge as BMI increased significantly. This result was consistent with Haas and colleagues’ study which found that leptin levels were significantly increased in patients with AN after weight restoration [36]. Leptin was labeled as the “satiety hormone,” which was released by fat cells located in the adipose tissue to inform the central nervous system by inducing a sensation of satiety through the hypothalamus to inhibit hunger [54,55]. The level of leptin was closely correlated with an individual’s percentage of body fat [56]. Upon admission, AN patients were underweight and malnourished. When the patients were discharged, they gained weight and had an increased BMI, which resulted in a rise in leptin levels. At 3 months post-discharge, leptin levels continued to rise despite a small rise in BMI.

Finally, cortisol levels at admission and discharge were both positively associated with the number of SBE episodes during the past four weeks at 3 months post-discharge, meaning that the higher the cortisol levels at admission or discharge, the more SBE episodes that were reported at 3 months post-discharge. Our results are inconsistent with Radin and colleagues’ experimental study, which found no significant association between cortisol levels and loss of control during eating among healthy adolescents [57]. To our knowledge, this is the first study that illustrates the significant relationship between cortisol levels and SBE. This relationship may be explained by the brain’s motivational systems. Neuroscience research on substance use suggests that the prefrontal cortex might influence cortisol secretion during times of stress, as well as self-control behaviors [58,59]. The hypothalamus regulates the HPA and controls the secretion of cortisol. However, during times of stress (possibly such as undergoing inpatient weight restoration treatment), the prefrontal cortex and limbic system take over cortisol regulation [59]. The prefrontal cortex plays a significant role in a wide variety of executive functions, including impulse control and self-control behaviors [60]. A key feature of SBE is the feeling of losing control during eating. Although a previous brain activity study using functional magnetic resonance imaging (fMRI) found that prefrontal cortex activity only varied with self-reported stress but not with cortisol levels [61], our findings suggest that cortisol might serve as an early marker of the feeling of losing control produced by the prefrontal cortex under stressful conditions. It is worth noting that both SBE and OBE have the component of loss of control during eating, but our results show no significant association between cortisol levels and OBE at 3 months post-discharge. The non-significant association between cortisol levels and OBE might be explained by the small sample size in our study (see post hoc power analyses in the limitations section). SBE is proposed as an important and clinically concerning characteristic of binge eating (regardless of the quantity consumed) and as a stronger predictor of eating disorder psychopathology and psychological disturbance than OBE [10,13,62]. It is noted that the recent version of the International Classification of Diseases-Eleventh Revision (ICD-11) eliminated the requirement that binge-eating episodes be defined by an unusually large amount of food, which officially recognizes the clinical importance of SBE based on increasing evidence suggesting that SBE (i.e., the sense of loss of control while eating) is more important than the amount of food consumed during binge-eating episodes [63]. Notably, we assessed OBE with a self-report questionnaire: the EDE-Q. Although the EDE-Q provided guidance as to how an OBE was defined, the item was still left up to interpretation by the participants. Considering our participants’ history of food restriction, their sense of what was objectively/unusually large might not map onto what trained assessors would call objectively/unusually large. Furthermore, during our inpatient treatment, participants did not have snacks or meals in addition to their regular meals and snacks. Therefore, it was unlikely that our participants could consume an objectively/unusually large amount of food when they stayed at the inpatient unit. Patients were also supervised by unit staff during all meals and snacks, as well as during bathroom use to prevent purging behavior.

This study should be considered within the context of its limitations. First, the current study contains a small sample size, which limits its statistical power to identify significant relationships between stress and appetite hormones and binge-eating episodes. We performed post hoc power analyses using nQuary (version 8.0), based on multiple linear regression coefficient estimates as effect sizes (Table 2 and Table 3), to evaluate the power of the multiple linear regression models [64]. Our sample size for this study was sufficient to analyze the relationships between admission or discharge cortisol levels and SBE episodes at 3 months post-discharge with a power of 82.63% (effect size = 0.37, α = 0.05, *n* = 55) and 77.08% (effect size = 0.36, α = 0.05, *n* = 51), respectively. For the non-significant relationships between admission or discharge hormone levels and the OBE or SBE episodes at 3 months post-discharge, the power value was only between 5.05% and 23.40 % (effect size = −0.01~0.16, α = 0.05, *n* = 45 ~ 68) to analyze the relationships. Additionally, we had only 5.05 to 18.62% power (effect size = −0.10~0.15, α = 0.05, *n* = 45~59) to examine the relationships between the change in hormone levels and OBE or SBE episodes. Second, some participants (*n* = 18) self-reported their body weight at 3 months after discharge, and this could affect the accuracy of weight measurements. We were also not able to obtain hormone measurements from these individuals at 3 months post-discharge. Third, single measures of cortisol and ACTH may not be the best indicators of the HPA axis activity because of the circadian rhythm of these two hormones [65]. In the current study, we only measured cortisol and ACTH levels at a single time point in a day, and the results may not represent changes in the hormones’ circadian rhythm when individuals were under psychological stress. Additionally, the hormones that we measured in the present study could be affected by situational factors. Studying the biological predictors of binge eating through experimental designs in a controlled environment in the future will help us to better understand the underlying mechanisms of binge eating.

Despite the limitations, our findings indicate changes in cortisol, total ghrelin, and leptin levels over time from admission through 3 months post-discharge and a positive relationship between cortisol levels (at admission and discharge) and SBE episodes in females with AN after weight restoration. Our findings could lead to identifying prospective biomarkers of binge-eating behaviors in AN patients after weight restoration. This work encourages the further study of the heterogeneity of AN such that interventions can be appropriately personalized based on behavioral characteristics and biomarkers, ultimately to improve the outcomes of AN.

## 5. Conclusions

Cortisol level might serve as an early indicator of loss of control during eating among females with AN after weight restoration. This highlights the importance of further research to (1) study the role of cortisol in predicting the loss of control during eating in a large sample, and (2) uncover the underlying mechanisms of loss of control during eating, cortisol, and brain activity. Such information could be used to develop prevention strategies, which not only prevent loss of control during eating, but also its negative consequences.

## Figures and Tables

**Table 1:** Demographic, clinical, and hormone variables for admission, discharge, and 3 months post-discharge.

Variables	Admission (Time 1)			Discharge (Time 2)			3 Months Post-Discharge (Time 3)					
	Mean	SD	*N*	Mean	SD	*N*	Mean	SD	*N*	χ^2 †^	*p* ^†^	Post hoc ^‡^
Demographics												
Age (year)	29.9	13.7	73	-	-	-	-	-	-	-	-	-
Age of onset (year)	17.8	7.2	72	-	-	-	-	-	-	-	-	-
BMI (kg/m^2^)	15.1	1.8	73	17.1	1.5	71	17.9	2.4	49	59.3	<0.01	T2 > T1 **
												T3 > T1 **
												T3 > T2 **
% IBW	68.8	7.8	73	79.5	5.7	71	83.5	10.9	49	67.8	<0.01	T2 > T1 **
												T3 > T1 **
												T3 > T2 **
Psychological factors												
Anxiety	25.1	13.3	67	18.6	11.7	64	17.7	11.1	50	16.9	<0.01	T1 > T2 **
												T1 > T3 **
Depression	32.9	15.7	65	22.1	16.5	63	21.6	15.3	49	17.8	<0.01	T1 > T2 **
												T1 > T3 **
Binge-eating												
OBE episode	13.9	52.6	72	4.3	13.0	73	2.4	5.9	73	6.8	0.03	T1 > T3 **
												
SBE episode	16.7	39.6	73	9.0	18.8	73	4.5	8.0	73	20.3	<0.01	T1 > T2 *
												T1 > T3 **
Hormones												
Cortisol (ug/dL)	19.9	6.4	55	19.1	7.1	51	14.8	6.4	24	6.3	0.04	T1 > T3 **
												T2 > T3 *
ACTH (pg/mL)	84.0	25.8	55	89.2	28.4	51	74.8	25.4	24	4.9	0.09	-
NE (pg/mL)	414.0	311.5	51	349.0	276.4	48	567.6	462.8	22	1.7	0.43	-
Ghrelin active (pg/mL)	216.5	104.0	68	192.9	75.5	60	273.6	184.2	28	1.7	0.44	-
Ghrelin total (pg/mL)	2153.0	1151.1	69	1360.6	558.8	61	1972.5	856.1	29	7.5	0.02	T1 > T2 **
												T3 > T2 *
Leptin (ng/mL)	8.4	5.7	53	15.3	13.4	45	21.1	18.3	17	8.1	0.02	T2 > T1 **
												T3 > T1 *

Note. BMI = body mass index; % IBW = the percentage of ideal body weight; OBE = Objective binge-eating episode during the past four weeks; SBE = Subjective binge-eating episode during the past four weeks; SD = standard deviation; ACTH = adrenocorticotropic hormone; NE = norepinephrine; Anxiety = sum score of the Beck Anxiety Inventory; Depression = sum score of the Beck Depression Inventory-II; ** *p* < 0.01; * *p* < 0.05; ^†^ Repeated-measures ANOVA on ranks; ^‡^ Wilcoxon Signed Rank Test with Bonferroni adjustment.

**Table 2:** Multiple linear regression results for associations of hormone levels at admission and discharge with objective and subjective binge-eating episodes at 3 months post-discharge.

	Objective Binge Eating ^a^				Subjective Binge Eating ^b^			
Admission	*n*	*β*	95% CI	*R* ^2^	*n*	*β*	95% CI	*R* ^2^
Cortisol (ug/dL)	55	0.08	(−0.18, 0.34)	0.01	55	0.37 *	(0.01, 0.72)	0.14
ACTH (pg/mL)	55	0.02	(−0.05, 0.09)	0.01	55	−0.02	(−0.11, 0.08)	0.07
NE (pg/mL)	51	<−0.01	(−0.01, 0.01)	0.01	51	<−0.01	(−0.01, 0.01)	0.07
Ghrelin active (pg/mL)	67	<0.01	(−0.01, 0.02)	0.01	68	0.01	(−0.01, 0.03)	0.10
Ghrelin total (pg/mL)	68	<0.01	(−0.001, 0.001)	<0.01	69	<0.01	(−0.002, 0.002)	0.09
Leptin (ng/mL)	53	0.16	(−0.18, 0.50)	0.03	53	−0.15	(−0.57, 0.27)	0.09
Discharge	*n*	*β*	95% CI	*R* ^2^	*n*	*β*	95% CI	*R* ^2^
Cortisol (ug/dL)	51	0.14	(−0.12, 0.40)	0.03	51	0.36 *	(0.03, 0.69)	0.15
ACTH (pg/mL)	51	0.03	(−0.03, 0.10)	0.02	51	0.02	(−0.07, 0.10)	0.06
NE (pg/mL)	48	<−0.01	(−0.01, 0.01)	0.03	48	<−0.01	(−0.01, 0.01)	0.07
Ghrelin active (pg/mL)	59	−0.01	(−0.02, 0.02)	<0.01	60	0.01	(−0.02, 0.03)	0.07
Ghrelin total (pg/mL)	60	<0.01	(−0.001, 0.004)	0.03	61	<0.01	(−0.01, 0.01)	0.08
Leptin (ng/mL)	45	<−0.01	(−0.16, 0.14)	<0.01	45	−0.13	(−0.33, 0.07)	0.10

Note. ^a^ Covariate: admission BMI, admission OBE; ^b^ Covariate: admission BMI, admission SBE; ACTH = adrenocorticotropic hormone; NE = norepinephrine; *β* = multiple linear regression coefficient estimate; 95% CI = 95% confidence interval; *R*^2^ = coefficient of determination; * *p* < 0.05.

**Table 3:** Multiple linear regression results for associations of changes in hormone levels with objective and subjective binge-eating episodes at 3 months post-discharge.

	Objective Binge Eating ^a^				Subjective Binge Eating ^b^			
Change from Admission to Discharge	*n*	*β*	95% CI	*R* ^2^	*n*	*β*	95% CI	*R* ^2^
Cortisol (ug/dL)	51	−0.10	(−0.41, 0.21)	0.01	51	−0.15	(−0.56, 0.26)	0.07
ACTH (pg/mL)	51	−0.02	(−0.10, 0.06)	0.01	51	−0.03	(−0.14, 0.07)	0.07
NE (pg/mL)	48	<0.01	(−0.004, 0.009)	0.02	48	<0.01	(−0.01, 0.01)	0.06
Ghrelin active (pg/mL)	58	<0.01	(−0.01, 0.02)	0.01	59	<0.01	(−0.01, 0.02)	0.08
Ghrelin total (pg/mL)	58	<0.01	(−0.002, 0.001)	<0.01	59	<0.01	(−0.002, 0.003)	0.08
Leptin (ng/mL)	45	0.03	(−0.16, 0.23)	<0.01	45	0.14	(−0.12, 0.40)	0.09

Note. ^a^ Covariate: admission BMI, admission OBE; ^b^ Covariate: admission BMI, admission SBE; ACTH = adrenocorticotropic hormone; NE = norepinephrine; *β* = multiple linear regression coefficient estimate; 95% CI = 95% confidence interval; *R*^2^ = coefficient of determination.
